# Outcomes of patellofemoral joint arthroplasty: a systematic review of revision timelines and complication rates

**DOI:** 10.1186/s13018-025-05592-8

**Published:** 2025-03-17

**Authors:** Martinique Vella-Baldacchino, Dean Chughtai, Jonathan Kow, Charlotte Carr, Amelie Coyle, Amerlia Farrow-Foster, Jemima Russell, Alexander D. Liddle

**Affiliations:** Department of Surgery and Cancer, MSk Lab – Imperial College London, Sir Michael Uren Hub, 86 Wood Ln, London, W12 0BZ UK

## Abstract

**Introduction:**

This systematic review attempts to address survivorship of patellofemoral joint replacements, with conversion to a total knee arthroplasty (TKA) as an endpoint.

**Methods:**

Survival estimates from multiple case series and national registries were pooled to calculate survival at 5, 10, 15 and 20 years, respectively. As a secondary outcome, the type and number of complications were recorded. A complication was defined as any any adverse event or unintended outcome that occurs as a result of the joint replacement, either during the immediate postoperative period or over the longer term. As arthroplasty registries do not report this information, this data was only included from publicly available series.

**Results:**

1015 eligible articles were identified, with 21 reporting survival and reasons for revision or complications. Data from registries were extracted. Using publicly available results from international joint registries, survival at 5 and 10 years were 90.30% (95% CI 88.32 to 92.27) and 82.23% (95% CI 78.90 to 85.56), respectively. However, long-term survivorship decreased to 73.74% (95% CI 69.12 to 78.37) and 72.68% (95% CI 69.58 to 75.53) at 15 and 20 years.

**Conclusion:**

Our pooled data, survival data from case series show similar results to international joint registries up to 10 years with a survival rate of 82.33%. These findings will be of use to patients and arthroplasty surgeons who require further information in order to predict how long patellofemoral joint replacements will last.

**Supplementary Information:**

The online version contains supplementary material available at 10.1186/s13018-025-05592-8.

## Introduction

PFJ osteoarthritis is the second most prevalent radiographic pattern of osteoarthritis and has a higher incidence in middle-aged women (24%) [1–3]. Patellofemoral joint replacements are a surgical treatment option for isolated patellofemoral joint osteoarthritis. This procedure preserves the cruciate ligaments of the knee and the intact cartilage in the tibiofemoral compartments whilst preserving joint proprioception and minimising the resection of healthy bone [4–7]. It is regarded as a bone and ligament-sparing procedure that offers the additional benefits of reduced blood loss, shorter operating times, and a faster recovery [8]. Despite the advantages of PFRs, they are linked to a high revision rate, with a reported 9.8% risk of revision within five years, which may explain why PFA usage remains at 1% in international joint registries [9–12]. Revisions of PFAs can be secondary to early complications (normally due to patella maltracking, subluxation, dislocation, or instability) or in the mid to long term due to progression of osteoarthritis [13, 14].

Patellofemoral joint repalcements have been described as onlay or inlay designs, these are based on the trochlea preparation method [15]. An onlay design is defined as a trochlea cutting design, which removes the trochlea surface using the same anterior femoral cuts as a total knee arthroplasty [16, 17]. Inlay designs are defined as trochlea resurfacing whereby the implants are embedded within the femoral bone without significantly changing the femoral shape [16, 18]. Nowadays, inlay implants such as the Richards II have been mostly abandoned and onlay implants are mainly used worldwide. Seven decades since the first prosthesis, patellofemoral joint replacements have undergone several design changes, with second-generation PFRs—devices developed at the beginning of the 1990s—showing promising results [19–21]. The implants used in this study are mainly the AVON Patellofemoral Joint Replacement (Stryker Howmedica Osteonics, Mahwah, NJ) which features a broad, symmetric trochlear flange and a medialized offset dome, designed to enhance patellar tracking [21, 22]. In contrast, the Zimmer Gender Solutions PFJ (Zimmer, Warsaw, IN) incorporates an asymmetric design that optimizes patellar tracking and reduces the need for lateral release during surgery [21]. The HemiCAP Inlay Resurfacing (Arthrosurface, Inc., Franklin, MA, USA) employs a stud that interlocks into the trochlea, utilizing a modular polyethylene component to avoid trochlear resection [23]. Lastly, the Journey PFJ (Smith & Nephew, Andover, MA) features an anatomical design with an asymmetric trochlear groove that is both deepened and lateralized, further enhancing patellar tracking [24].

This study investigates how long patellofemoral joint arthroplasties last using all of the patellofemoral joint arthroplasty series published to date and data from all national joint registries. Secondary outcome measures included an overview of the complications sustained following this procedure.

## Methods

### Search strategy and selection criteria

A predefined protocol was registered on Prospero and adhered to using PRISMA guidelines. A search was conducted using MEDLINE, EMBASE via OVID, CINAHL, and EBSCO, searching for case series and cohort studies that reported the survival outcomes of patellofemoral arthroplasties, published between the start of the database and January 27, 2025. The search strategy may be found in the. Studies included adult patients > 18 who underwent a patellofemoral joint arthroplasty for end stage degenerative osteoarthritis. Systematic reviews were retrieved, and citations were searched; however, systematic review data were excluded to avoid duplication.

The primary outcome of this study was to understand how long patellofemoral joint arthroplasties last, defined as a revision for any reason. The term revision was defined as the time interval between the date of the initial surgery and the date of the revision where all components were removed. Each prosthesis’s mean or median survival was recorded at 5, 10, and 15 years. The closest time point was taken if data was collected at any other time. The survival outcomes from all international arthroplasty registries were also determined from publicly available annual reports, which collect data on all patellofemoral joint arthroplasties in both the public and private sectors. Reasons for revision were extracted. As a secondary outcome in case series and cohort studies, the type and number of complications were recorded in each study. A complication was defined as any any adverse event or unintended outcome that occurs as a result of the joint replacement, either during the immediate postoperative period or over the longer term. Scientific reports of arthroplasty registries do not include this information.

### 2.2 Abstract screening and data extraction

Three reviewers (DC, JK, MVB) screened the abstracts of all articles using the web application Covidence. Any disagreements between the reviewers were resolved with the involvement of a third person (MVB). The two reviewers (DC, JK) independently extracted data using a specifically designed standardised data extraction form on Excel (Microsoft, USA), and the extracted data was compared afterwards for consistency. All discrepancies were resolved through discussion between the two data extractors. For each included study, the total number of knees and patients, gender and type of implant, mean or median survival and number and type of complications were recorded. The authors were contacted to provide this information if complete data from full-text articles were unavailable. The study was excluded from data synthesis if the authors did not respond following a subsequent email.

### Data analysis

**Fig. 1:**
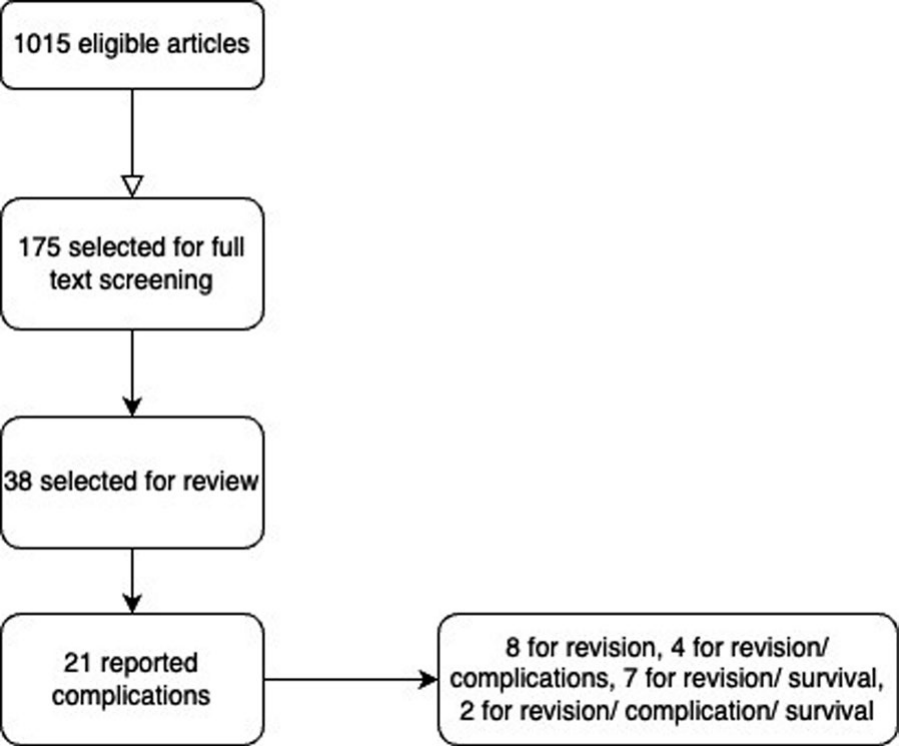
Flowchart of studies review and included for analysis. This flowchart shows the process of identifying, screening and selecting studies for the analysis

Patellofemoral joint arthroplasty survival estimates at 5, 10 and 15, 20 years were pooled into forest plots using sample size and confidence intervals. All statistical analysis was performed using Stata (Stata 18, Houston, Texas) to produce forest plots and R software (R version 4.3.3, Foundation for Statistical Computing, Vienna, Austria) to visualise survival percentages over time (see Fig. 1). Each study was weighted according to its standard error, calculated from published confidence intervals. A random effect model was used. Data from registries were pooled into forest plots in the same way. The code is available in the online Appendix section.

### Study quality

A risk of BIAS assessment using ROBINS-I for non-randomised interventions (Supplementary Table 5). Three independent coders (CC, AC, AFF,JR) assessed study quality, and disagreements were resolved via discussion to reach a final decision. The studies were then classified into overall low, medium, and high based on the scoring protocol of the instruments.

## Results

**Fig. 2:**
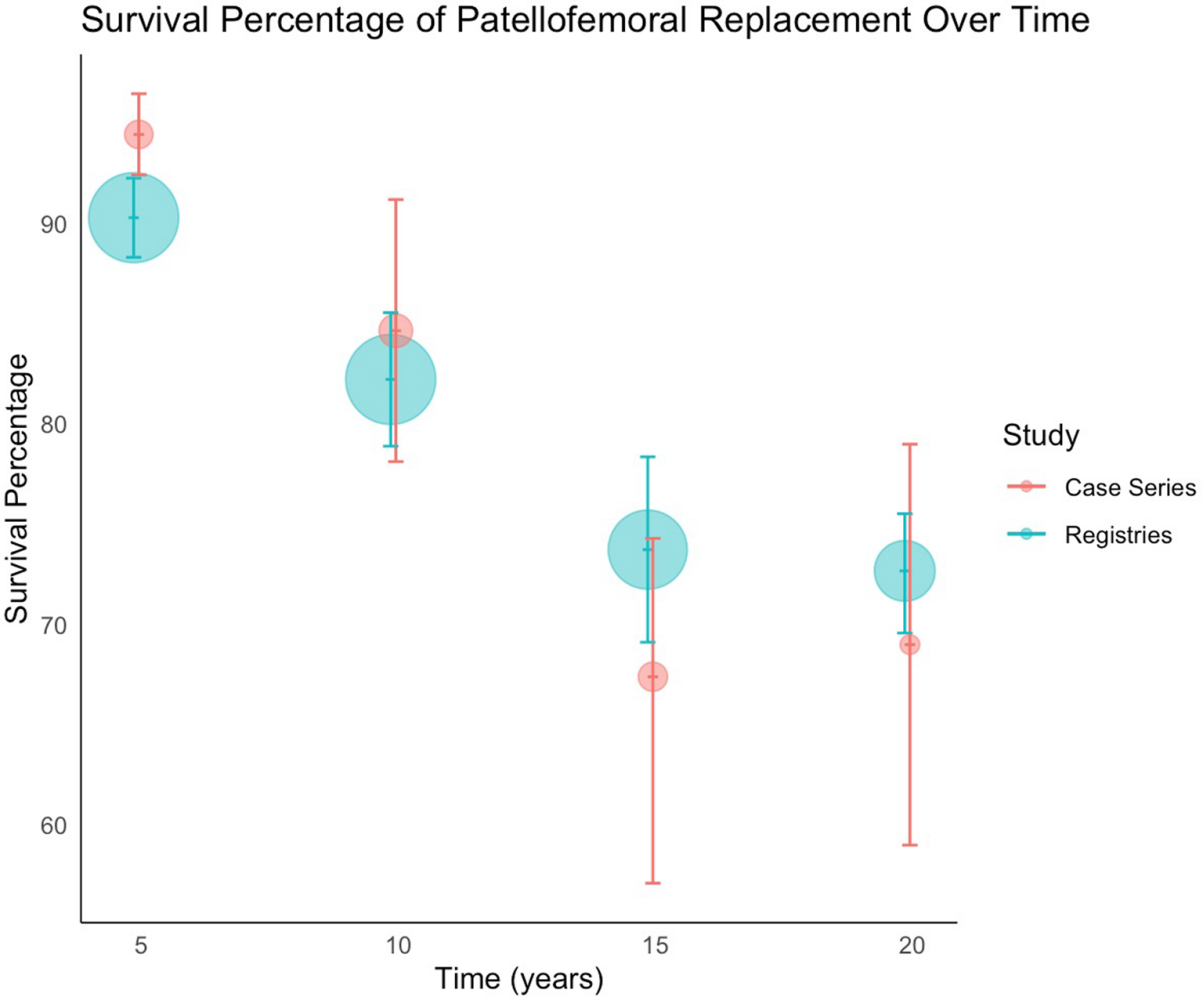
Forest plot with pooled results from case series and registries. This forest plot shows the combined results for survival from case series and registries. Confidence intervals and relative weighting of results, calculated according to study size, are also shown

**Fig. 3:**
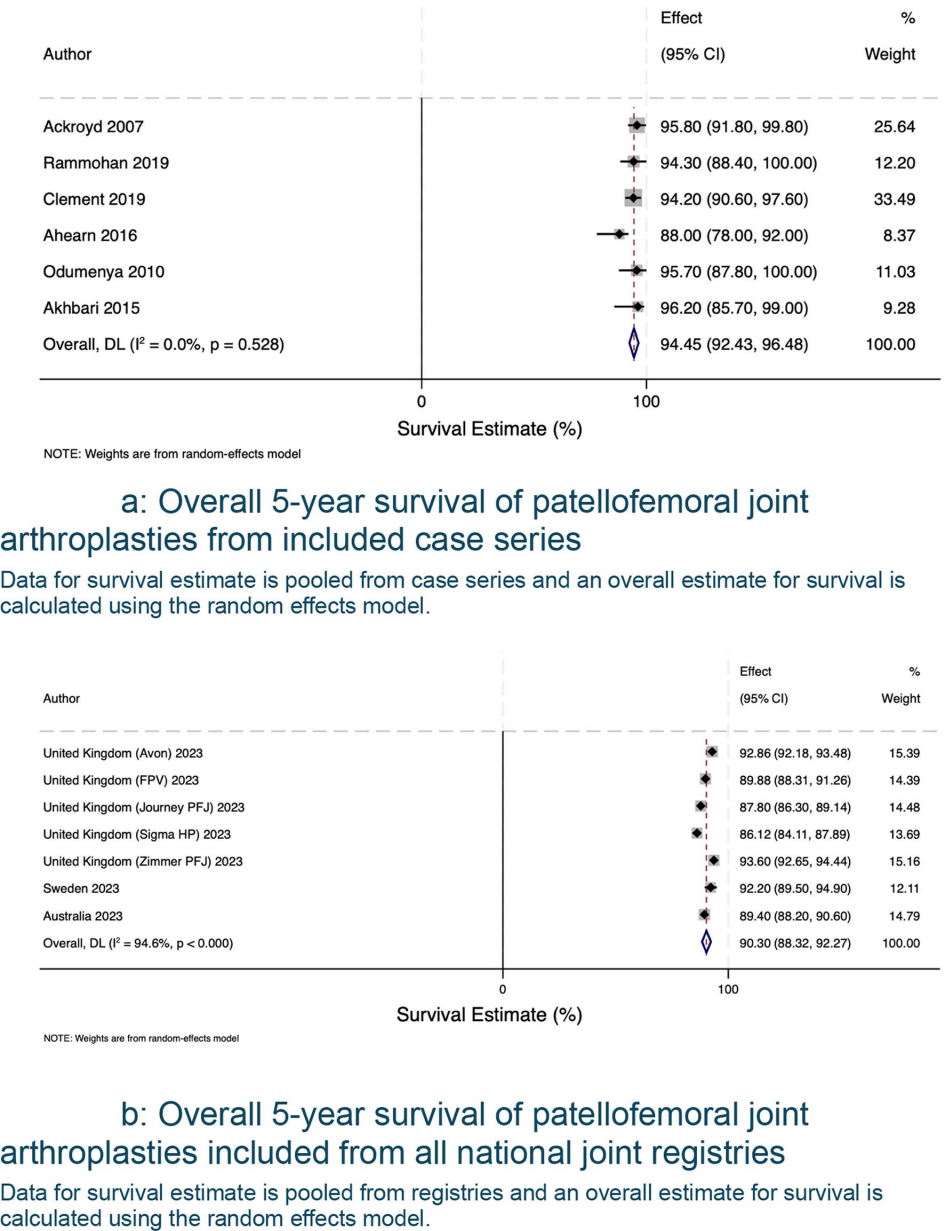
**a** Overall 5-year survival of patellofemoral joint arthroplasties from included case series. Data for survival estimate is pooled from case series and an overall estimate for survival is calculated using the random effects model. **b** Overall 5-year survival of patellofemoral joint arthroplasties included from all national joint registries. Data for survival estimate is pooled from registries and an overall estimate for survival is calculated using the random effects model

**Fig. 4:**
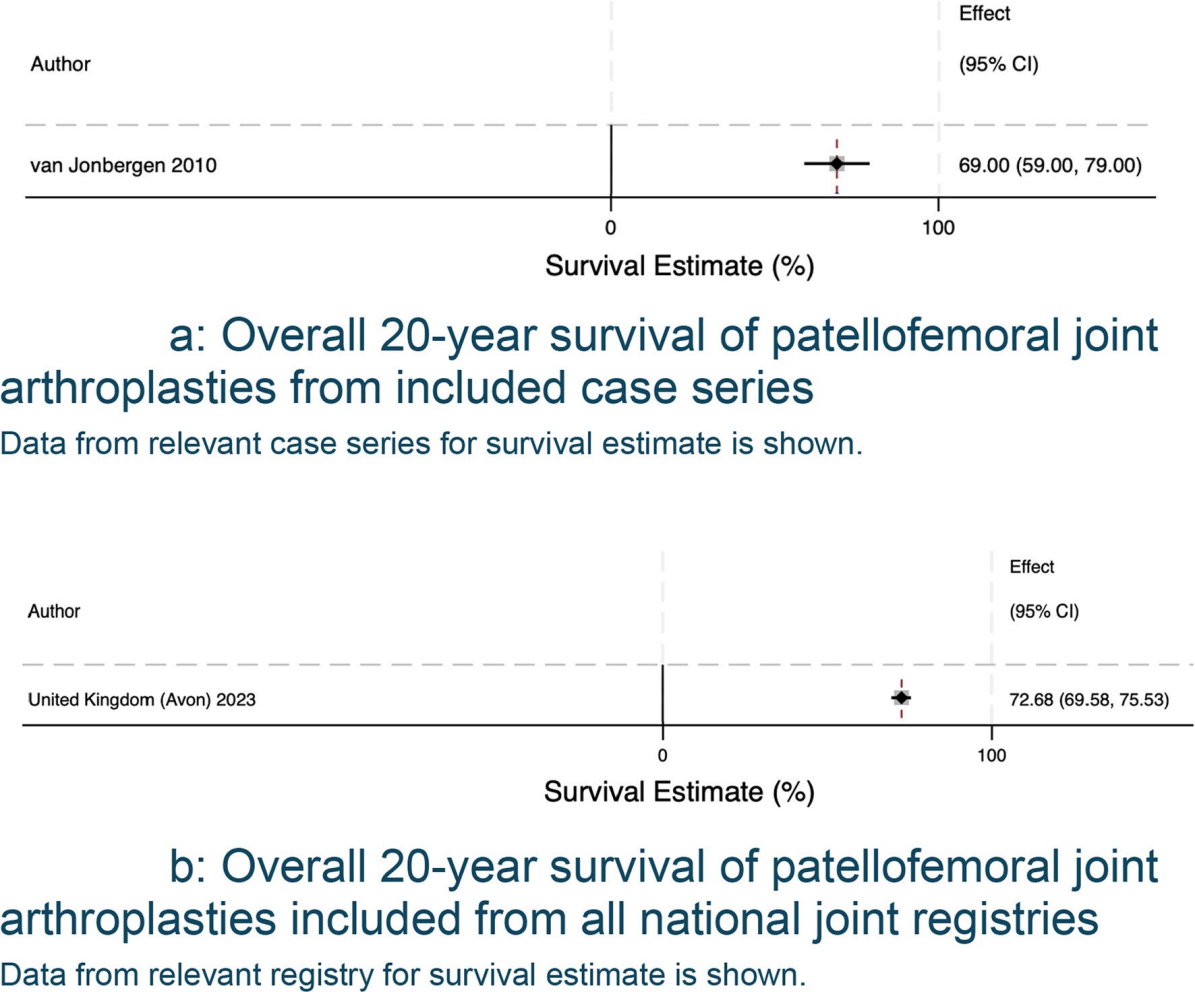
**a** Overall 20-year survival of patellofemoral joint arthroplasties from included case series. Data from relevant case series for survival estimate is shown. **b** Overall 20-year survival of patellofemoral joint arthroplasties included from all national joint registries. Data from relevant registry for survival estimate is shown

The search of published case series produced 1015 eligible articles, with 175 proceeding to full-text screening, 8 articles selected for review, and 21 articles with data available for extraction and analysis. A summary of included articles and registries can be found in Table 1. There was a total of 21 unique case series, reported a total of 2181 patellofemoral joint arthroplasties (range 28–483 PFRs). Table 2 summarises each data source’s study level, patient-level characteristics and surgical experience. The average age was 59.1, the cohort included more female patients, for every male patient, there were three female patients. In terms of surgical experience, most authors did not report the background of the operating surgeons, mentioned the number of individuals involved without further detail, or described them as experienced without defining what ‘experienced’ entails. Pooled analysis of data derived from case series and registries is shown in Fig. 2. The forest plot shows survivorship of over 90.30% (95% CI 88.32 to 92.27) at five years from both published case series and registry data (Fig. 3a, b). This decreases to 72.68% (95% CI 69.58 to 75.53) at 20 years (Fig. 4a, b). Pooled forest plots for data included from case series and national joint registries at 5 years, 10 years, 15 years and 20 years.

**Table 1:** A summary of individual case series and data included from international joint registries

	Individual Case Series	National Joint Registry annual report, 2023	Australian Orthopaedic Association National Joint Replacement Registry annual report, 2023	Swedish Arthroplasty Register annual report, 2023
Location	8 Countries	United Kingdom	Australia	Sweden
Number of Unique Series Included	21 articles	5	Unknown	Unknown
Year of Publication	2007–2023	2023	2023	2023

**Table 2:** Summary of individual case series included in the study

Study ID	Number of Knees Included	Number of Knees at Final Follow Up	Final Follow Up	Implant	% Male of original cohort	Average Age	Survival %	Survival given at	Reason for Inclusion	Robins-I	Surgeon Experience
Ackroyd et al. 2007	109	83	5 Years (5–8)	Avon	11.76 (10/85)	68 (46–86)	95.8 (91.8–99.8)	5 Years	Complications + Survival	Critical + Critical	More than 20 consultants and trainees
Rammohan et al. 2019	103	102	6 Years (2–11)	Journey	31.64 (25/79)	58 (42–78)	94.3 (88.4–100)	7 Years	Complications + Survival	Critical + Critical	Not reported
Leadbetter et al. 2009	79	79	3 Years (2–6)	Avon	25.71 (18/70)	58 (34–77)	Not reported	N/A	Complications	Critical	Not reported
Imhoff et al. 2019	37	24	5 Years (2–5)	HemiCAP	68.57 (24/35)**	49 (22–79)	83	5 Years	Complications	Serious	Not reported
Middleton et al. 2018	103	103	6 Years (3–14)	Avon	34.95 (36/103)	60 (34–82)	89	5 Years	Complications	Critical	Not reported
							86	10 Years			
Wang et al. 2023	46	28	24 Years (19–30)	YLQ	13.16 (5/38)	55 (36–73)	83.6	15 Years	Complications	Critical	Not reported
							76.8				
								20 Years			
							59.4				
								25 Years			
Clement et al. 2019	54	54	10 Years (8–15)	Avon	9.26 (5/54)	62 (Not reported)	94.2 (90.4–98.1)	5 Years	Complications + Survival	Critical + Critical	Not reported
								10 Years			
							92.3 (87.1–97.5)				
Metcalfe et al. 2018	558	390	18 Years*** (2–18)	Avon	18.41 (79/429)	59 (25–92)	77.3 (72.4–81.7)	10 Years	Complications + Survival	Critical + Serious	Not reported
								15 Year			
							67.4(57.1–74.3)				
Hoogervorst et al. 2015	33	28	6 Years (2–18)	Richards’ II	20.83 (5/24)	47 (32–81)	73 (57–93)	10 Years	Complications + Survival	Critical + Critical	Not reported
Akhbari et al. 2015	61	55	5 Years (1–10)	Avon	10.53 (6/57)	66.1 (Not reported)	96.2 (85.7–99.0)	5 Years	Complications + Survival	Critical + Critical	Not reported
								10 Years			
							88.9 (71.8–96.0)				
Ahearn et al. 2016	101	90	7 Years (5–9)	Journey	19.80 (20/101)	60 (26–86)	88 (78–92)	7 Years	Complications + Survival	Critical + Critical	Not reported
van Jonbergen et al. 2010	185	181	13 Years (2–30)	Richards’ II	37.58 (59/157)	52 (Not reported)	84 (78–90)	10 Years	Complications + Survival	Critical + Moderate	Not reported
							69 (59–79)	20 Years			
Odumenya et al. 2010	67	50	5 Years (2–10)	Avon	28.13 (9/32)	66 (42–88)	95.7 (87.8–100)	5 Years	Complications + Survival	Critical + Critical	Not reported
								8 Years			
							89.3 (72.9–100)				
Osarumwense et al. 2017	52	49	3 Years (2–5)	Zimmer	25.00 (9/36)	59 (39–80)	95.6	5 Years	Complications	Critical	Not reported
Romagnoli and Marullo 2017	108	105	6 Years (3–8)	Zimmer	18.82 (16/85)	68 (39–88)	95.2	6 Years	Complications	Critical	Not reported
Bohu et al. 2019	74	70	8 Years (2–20)	Hermes	18.75 (12/64)	60 (31–82)	85.7	8 Years	Complications	Critical	1 Senior Surgeon
deDeugd et al. 2017	75	75	3 Years (2–10)	Avon	12.00 (9/75)	51 (36–81)	94.7	5 Years	Complications	Critical	Not reported
Ramos et al. 2016	157	157	7 Years (7–7)	Avon or VanguardPF	26 (Not reported)	Not reported	Not reported	N/a	Complications	No Information	Not reported
Henrigou et al. 2014	85	70	12 Years (10–16)	Hermes	46.67 (28/60)	71 (Not reported)	Not reported*	N/a	Complications	Critical	Not reported
Konan and Haddad 2016	51	48	7 Years (5–11)	Avon	61.7 (29/47)	57 (37–69)	96.1	11 Years	Complications	Critical	Not reported
Mont et al. 2012	43	43	7 Years (4–8)	Avon	21.62 (8/37)	49 (27–67)	95	5 Years	Complications	Critical	3 Experienced Surgeons
							82	7 Years			

^**^only reports M:F knees at 2 year follow up

^***^ study only reports final follow up time, not average

The studies included were assessed for risk of bias using the Robins-I tool. Most studies were deemed to have a critical level of bias (Table 3) due to the lack of adjustment for confounding variables. However, the data was still included in the analysis as the nature of case series typically reports outcomes without statistical adjustment, paired with the limited literature available for patellofemoral arthroplasties (with > 1% of all arthroplasties being a PFR), which makes it necessary to use this data despite its limitations.

**Table 3:**
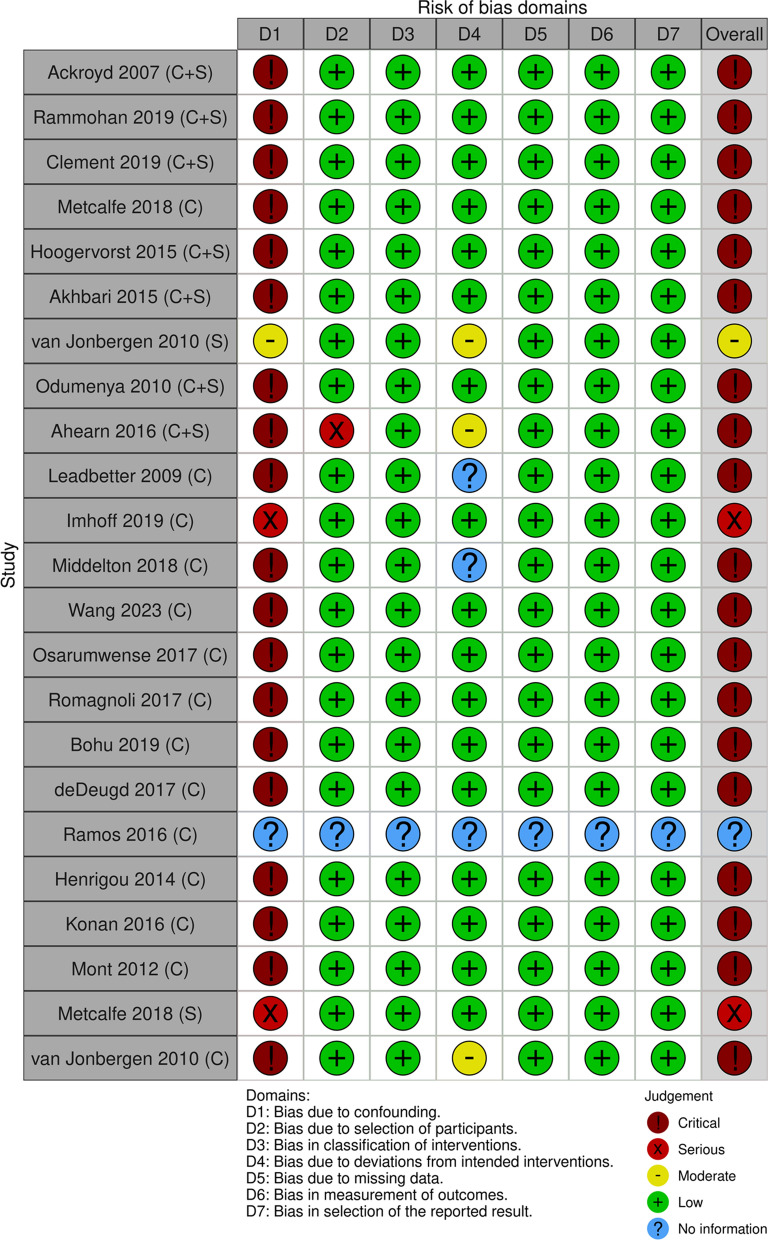
for risk of bias using ROBINS-I. ‘C’ denotes complication/reason for revision outcomes, and ‘S’ denotes survival analysis outcomes

Reasons for revision following patellofemoral joint arthroplasty surgery in the included case series are shown in Table 4. The most common reason for the revision was the progression of osteoarthritis and pain. For each paper, the grading of osteoarthritis and the scales used to measure pain were assessed. However, there was significant variation among authors, with few using the same scale or reporting the specific measurement tool utilized. All relevant data can be found in the appendix. A summary of local complications sustained following each case series can be found in Table 5. The most common complication was pain (14.97%), followed by arthrofibrosis (3.57%).

**Table 4:** Reasons for revision from all case series included in the study

Paper Author	Year	Knees	Prosthesis	Revisions Total	Revision Reason	Time to Revision
Leadbetter	2009	79	Avon	5	Progression of OA (4) Instability (1)	Not reported
Ackroyd	2007	83	Avon	4	Progression of OA (4)	End of study (5 years)
Rammohan	2019	103	Journey	4	Patellar maltracking (1) Persistent pain (1) Progression of OA (2)	1.5 years 2.5 years 4.5, 6 years
Imhoff	2019	35	Hemicap	6	Pain (5) Allergy leading to progressive synovitis (1)	2.3 ± 1.1 years
Middelton	2018	103	Avon	10	Progression of OA (9) Trochlear malpositioning (1)	2.9 years (1–6 years)
Wang	2023	28	YLQ prosthesis	14	Progression of OA (11)* Polyethylene wear (1)	16.0 ± 6.7 years
Clement	2019	54	Avon	5	Progression of OA (2) Pain (2) Fracture (1)	End of study (15 years) End of study (15 years) 6 years
Metcalfe	2018	483	Avon	105	Progression of OA (61) Pain (12) Femoral loosening (7) Button wear (6) Mal-alignment/ mal-sizing (2) Avascular necrosis (2) Unknown (18)	Not reported Not reported Not reported Not reported Not reported Not reported Not reported
Hoogervorst	2015	28	Richards’ II	12	Progression of OA (5) Infection (1) Pain (3) Instability (3)	3.1, 5.6, 5.7, 7, 7, 7.2 years 2.8 years 0.2, 11.8 years, not reported Not reported
Akhbari	2015	55	Avon	4	Progression of OA (3) Patella maltracking (1)	1.7, 5.3, 6.2 years 2.2 years
van Jonbergen	2010	181	Richards’ II	41	Progression of OA (23) Patella maltracking (10) Loosening (4) Wear (4)	11.7 years average
Odumenya	2010	50	Avon	3	Progression of OA (2) Unknown (1)	Not reported Not reported
Ahearn	2016	90	Journey	12	Progression of OA (8) Pain (1) Maltracking (1) Infection (1) Broken trochlear component (1)	2.3, 2.7, 2.7, 3.2, 4.8, 6.8, 7.3, 8.3 years 2.1 years 0.8 years 4.9 years 4.3 years
Osarumwense	2017	49	Zimmer Gender Solutions	2	Progression of OA (2)	2.2 and 2.4 years
Romagnoli	2017	105	Zimmer Gender Solutions	3	Progression of OA (1) Fall (1) Wrong Indication (1)	Not reported 3 years 2.4 years
Bohu	2019	70	Hermes	15	Progression of OA (9) Pain (2) Malposition (1) Cementing issue (2) Unknown (2)	5 years (1–9) 1, 18 years 1.2 years 2.2, 8.5 years Not reported
deDeugd**	2017	75	Avon	4	Specific reason for failure not reported (4)	Not reported
Ramos	2016	157	Avon or Vanguard PF	3	Pain or Progression of OA (3)	Not reported
Henrigou	2014	70	Hermes	3	Progression of OA (3)	7 years (4–10)
Konan	2016	48	Avon	2	Progression of OA (1) Pain (1)	Not reported Not reported
Mont	2012	43	Avon	5	Pain (3) Aseptic loosening (2)	Not reported 7, 8 years

**Table 5:** Summary of local complications following patellofemoral joint replacements

Complication	Patients %	Paper
Haematoma	0.97	Rammohan 2019
Patella Fracture	0.97	Rammohan 2019
Wound Infection	1.94 3.57	Rammohan 2019 Wang 2023
Maltracking	0.97	Rammohan 2019
Postoperative Haemarthrosis	2.41	Ackroyd 2007
Synovitis	1.20 2.86	Ackroyd 2007 Imhoff 2019
Delayed Wound Healing	2.41	Ackroyd 2007
Pain	6.33 14.29	Leadbetter 2009 Imhoff 2019
Arthrofibrosis/MUA	1.27 1.20 2.91 2.91 3.57	Leadbetter 2009 Ackroyd 2007 Rammohan 2019 Middleton 2018 Wang 2023
Tibial Tubercle Fracture	1.27	Leadbetter 2009

## Discussion

The study shows that patellofemoral joint arthroplasty survival decreases from 90% at five years to around 70–80% at 20 years, with progression of osteoarthritis as the most common reason for revision. This revision rate differs significantly from the first generation of patellofemoral joint arthroplasties designed before the 1990s [9, 17, 25]. The study results demonstrate significant advancements in surgical design and understanding, which have evolved from earlier reports of a 35% revision rate [26–28]. A key goal in patellofemoral joint replacement design is to achieve precise geometric mating between the patella and trochlea, as any mismatch can lead to component malalignment. Early design variations that failed to address this critical issue have long hindered the widespread adoption of patellofemoral arthroplasty since its introduction in 1955 [21, 29]

**Fig. 5:**
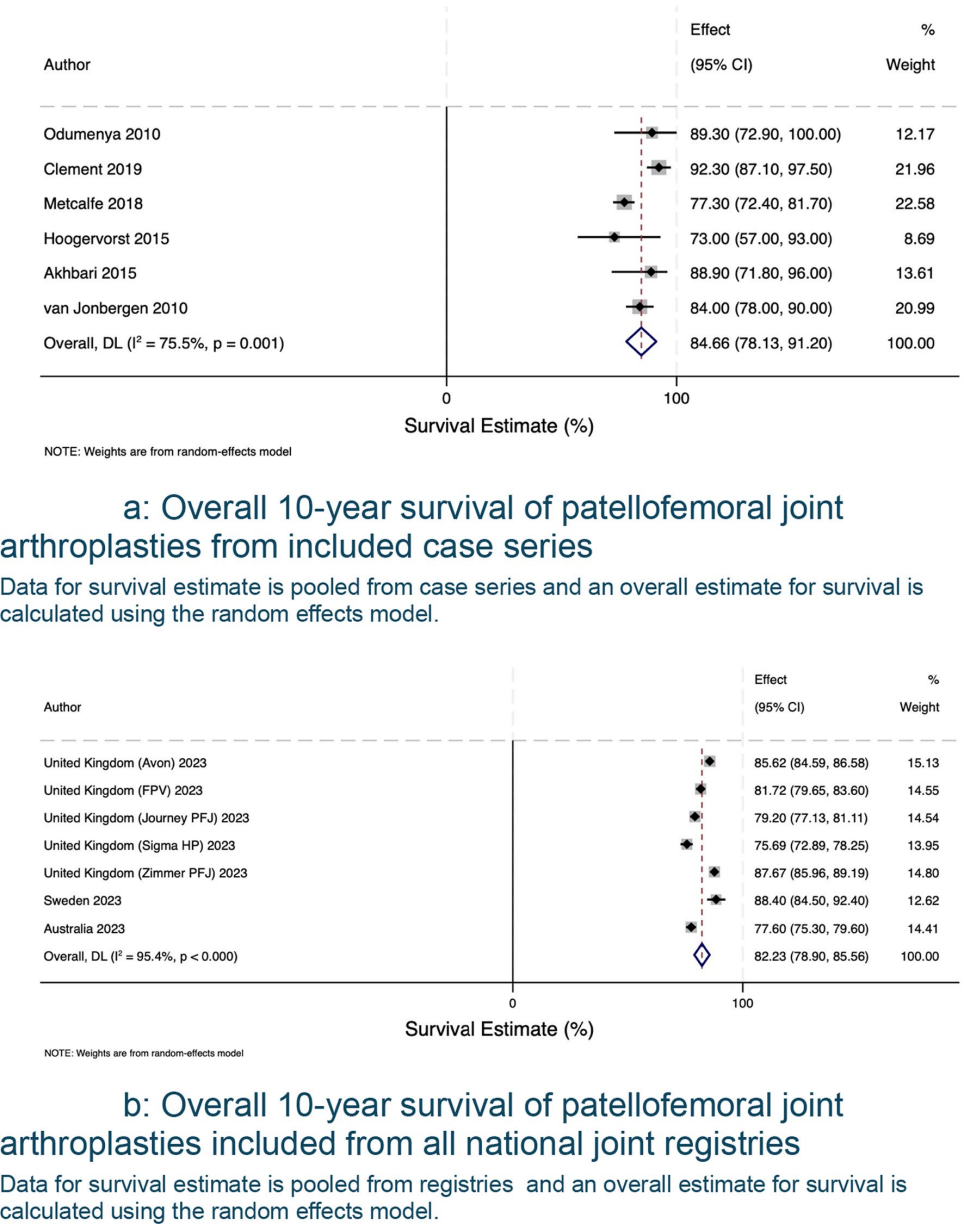
**a** Overall 10-year survival of patellofemoral joint arthroplasties from included case series. Data for survival estimate is pooled from case series and an overall estimate for survival is calculated using the random effects model. **b** Overall 10-year survival of patellofemoral joint arthroplasties included from all national joint registries. Data for survival estimate is pooled from registries and an overall estimate for survival is calculated using the random effects model

Compared to total knee arthroplasties, partial knee arthroplasties always have a higher revision rate [4, 30]. A revision of a patellofemoral joint arthroplasty should not be frowned upon since, in 83% of cases, a primary total knee arthroplasty was the revision procedure and using stems or augments was infrequent [5, 31]. Patellofemoral joint arthroplasties are recommended for patients between 40 and 60 [21]. This treatment option may be appropriate for working-age patients, as it provides patients with an 82.23% (95% CI 78.90 to 85.56) survival ten-year prosthesis survival rate (Fig. 5b). In addition, recent literature has shown that if the surgeon experience consists of more than 5 PFA per year, the revision rate significantly drops. Most of the papers included in this study did not consider surgeon experience as a factor [32].

Surgical results following TKA may be considered reproducible by surgeons due to the relatively high number of TKAs, 52 cases per surgeon per year versus 3.7 PFAs per surgeon per year[26]. Partial knee arthroplasty is associated with a higher revision rate compared to total knee arthroplasty, so it is reasonable for surgeons to prefer using a procedure that has a lower revision rate whilst concomitantly maintaining their low revision status by using a method that is less likely to require revision [2, 4, 15, 33].

This study showed that patellofemoral joint arthroplasty is a safe alternative; however, with shared decision-making, it should be acknowledged that the procedure is not foolproof, and the risk of revision increases over time. Additionally, PFA and TKA revision rates should be compared cautiously since patients and surgeons may have a lower threshold for revising a partial arthroplasty due to the perceived lower morbidity associated with PFA [5, 29]. The progression of osteoarthritis is the most common reason for revision, but as Table 5 shows, the most common complication was pain (14.97%), followed by arthrofibrosis (3.57%).

Further research is required to explore the causes of pain following patellofemoral joint replacements, as prior studies have demonstrated that patients experiencing pain are 2.5 times more likely to require revision surgery [34]. While some studies suggest that pain may results from joint overstuffing [11]. The use of robotic technology may prove helpful in addressing appropriate implant sizing and assisting surgeons without a high level of experience in determining an appropriate position and implant size [19, 24, 35, 36]. Addressing post-operative pain effectively may therefore help reduce revision rates.

Reviews on patellofemoral joint arthroplasties have been reported in various ways, whether comparing PFR and TKR using patient-reported outcomes or a study on the overall survival of just patellofemoral joint arthroplasty[12, 37–39]. Lonner et al. report patellofemoral joint arthroplasties’ survival rates, including case series with less than 4 years of follow-up [40]. The authors report that 0–35% of patellofemoral joint arthroplasties are revised [40]. Lewis et al. report patellofemoral joint arthroplasty survival using only international registry data, reporting an 8–18.1% revision rate at 5 years [5]. This study provides a significant contribution to the field of patellofemoral joint arthroplasty, for the first time, consolidating the results of all published case series and registry data on patellofemoral arthroplasty into a single comprehensive analysis. This approach offers surgeons and patients a unique and valuable resource: a graphical summary of the survival expectancy of this procedure. Unlike previous studies that report independent case series in isolation, this work serves as a definitive summary, offering an overarching perspective on revision rates and the most common complications. By presenting a holistic view, the study bridges a critical gap in the literature, enabling evidence-based decision-making and fostering a clearer understanding of the long-term outcomes associated with this procedure. All studies with over four years of survival data were included in the survival data. Studies were not categorised by implant type. However, this was not the paper’s aim and other published papers have reported this [40]. The authors could only include data from international joint registries, which published their data in publicly available annual scientific reports. One limitation of this study is the lack of registry data available. A total of 5 were screened, but only three were used. The American and New Zealand registry was excluded due to a lack of specific data for the analysis. Despite this, the relative heterogeneity between the included registries suggests that the data analysed is valid and the conclusions drawn are reliable.

## Conclusion

**Fig. 6:**
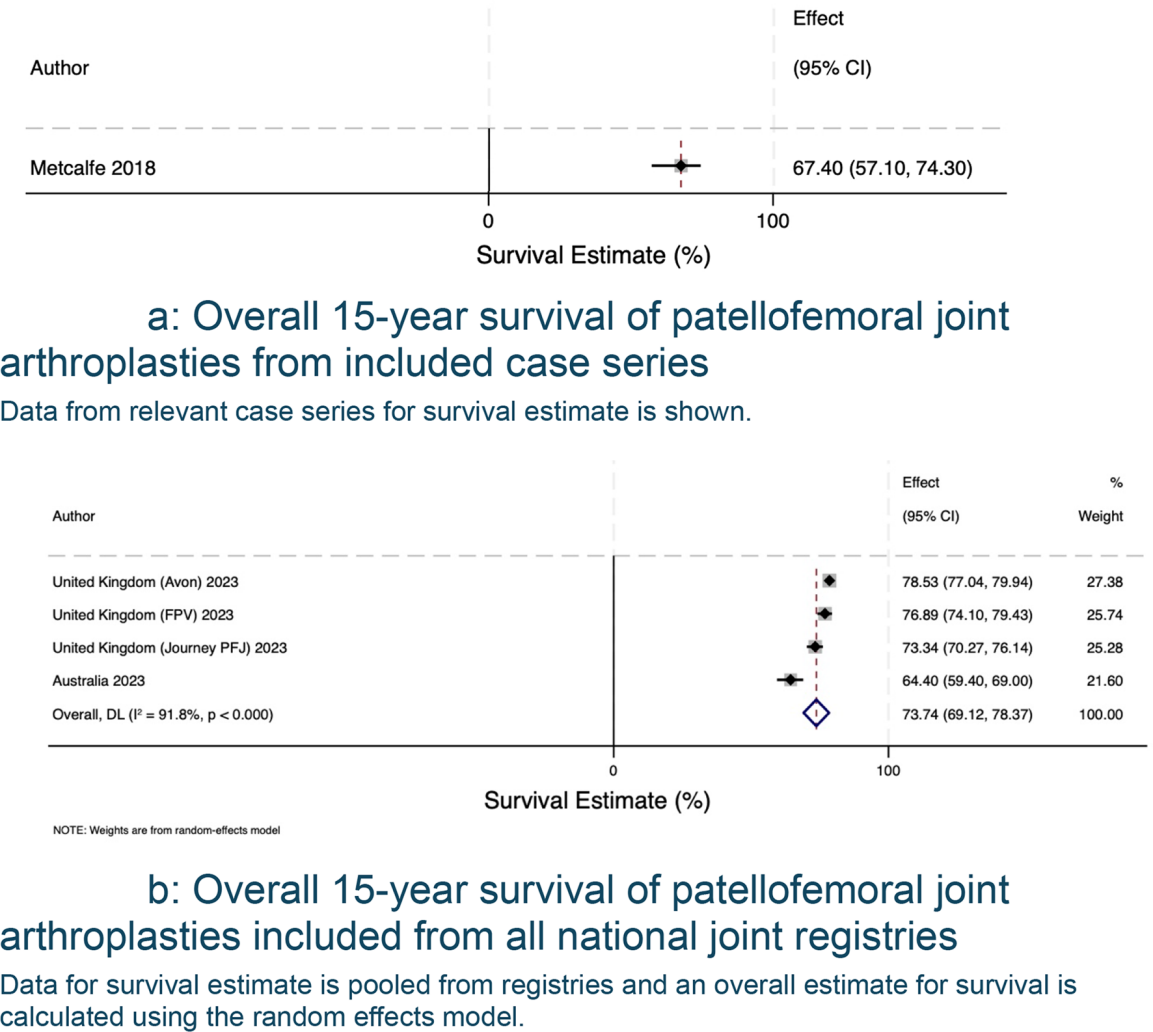
**a** Overall 15-year survival of patellofemoral joint arthroplasties from included case series. Data from relevant case series for survival estimate is shown. **b** Overall 15-year survival of patellofemoral joint arthroplasties included from all national joint registries. Data for survival estimate is pooled from registries and an overall estimate for survival is calculated using the random effects model

The pooled survival data from the case series show similar results to those from international joint registries up to 10 years. Beyond 10 years, the case series results are more optimistic than those from the national joint registry. Using the results from this study, patellofemoral joint arthroplasties have a survival rate of 82.23% (95% CI 78.90 to 85.56) at 10 years (Fig. 5b), decreasing to 72.68 (95% CI 69.58 to 75.53) (Fig. 6b) at 20 years if one considers the registry data as the more accurate data sample. The most common reason for revision was progression of osteoarthritis and pain was the most common complication.

## Supplementary Information

Below is the link to the electronic supplementary material.

Supplementary file 1.

## Data Availability

No datasets were generated or analysed during the current study.
