# Supplementary material for: Outcomes of patellofemoral joint arthroplasty: a systematic review of revision timelines and complication rates
Source: J Orthop Surg Res. 2025 Mar 17;20:289. doi: 10.1186/s13018-025-05592-8 (PMC11917147; doi:10.1186/s13018-025-05592-8)
Supplement: Supplementary file 1 [file 13018_2025_5592_MOESM1_ESM.docx]

# Appendix

| ***Paper Author*** | ***Year*** | ***Measure(s) for Pain*** | ***Additional Information*** |
| --- | --- | --- | --- |
| Rammohan | 2019 | OKS, Lysholm Score, Kujala Score, Bartlett Score | Degeneration and delamination of the lateral femoral condyle |
| Imhoff | 2019 | WOMAC and VAS | Anterior knee pain |
| Clement | 2019 | OKS and SF-12 | Not reported |
| Metcalfe | 2018 | OKS and WOMAC | Cause unknown |
| Hoogervorst | 2015 | KSS and VAS | Not reported |
| Ahearn | 2016 | OKS and WOMAC | Unexplained pain |
| Bohu | 2019 | AKP and IKS | Loosening without tibiofemoral arthritis |
| Ramos | 2016 | KOOS | Not reported |
| Konan | 2016 | OKS and VAS | Not reported |
| Mont | 2012 | KSS | Not reported |

WOMAC: Western Ontario and McMaster Universities Osteoarthritis Index

VAS: Visual Analogue Scale (for pain assessment)

OKS: Oxford Knee Score

SF-12: Short Form-12 Health Survey

KSS: Knee Society Score

AKP: Anterior Knee Pain Score

IKS: International Knee Society Score

KOOS: Knee Osteoarthritis Outcome Score

## Table A1: Pain Measurement Tools and Additional Information in Studies on Knee Outcomes

| ***Paper Author*** | ***Year*** | ***Measure for OA progression*** |
| --- | --- | --- |
| Leadbetter | 2009 | Not reported |
| Ackroyd | 2007 | No scale/classification used |
| Rammohan | 2019 | Not reported |
| Middelton | 2018 | Not reported |
| Wang | 2023 | Not reported |
| Clement | 2019 | Not reported |
| Metcalfe | 2018 | Not reported |
| Hoogervorst | 2015 | Not reported |
| Akhbari | 2015 | Not reported |
| van Jonbergen | 2010 | Not reported |
| Odumenya | 2010 | No scale/classification used |
| Ahearn | 2016 | Not reported |
| Osarumwense | 2017 | Outerbridge |
| Romagnoli | 2017 | Not reported |
| Bohu | 2019 | Not reported |
| Ramos | 2016 | Not reported |
| Henrigou | 2014 | Not reported |
| Konan | 2016 | Not reported |

## Table A2: Measures of Osteoarthritis (OA) Progression in Reviewed Studies

# Code used in Stata (Stata 18, Houston, Texas) to create forest plots

metan acconstructsurvival acconstructlowerci acconstructupperci if (acconstructtime==5 | acconstructtime==10 | acconstructtime==15 | acconstructtime==20) & implanttype==3, label(namevar=author)

# Code used in R software (R version 4.3.3, Foundation for Statistical Computing, Vienna, Austria) to create forest plot for pooled case series and registry data

# Load required libraries

library(ggplot2)

# Sample data (replace this with your actual data)

data <- data.frame(

Time = rep(c(5, 10, 15, 20), each = 2), # Time points

Study = rep(c("Registries", "Case Series"), times = 4), # Updated Study labels

Survival_Percentage = c(90.30, 94.45, 82.23, 84.66, 73.74, 67.4, 72.68, 69), # Example survival percentage

CI_Lower = c(88.32, 92.43, 78.90, 78.13, 69.12, 57.1, 69.58, 59), # Example lower confidence interval

CI_Upper = c(92.27, 96.48, 85.56, 91.2, 78.37, 74.3, 75.53, 79), # Example upper confidence interval

Size = c(20018, 495, 20018, 958, 14329, 558, 6952, 185) # Example study sizes

)

# Plot

ggplot(data = data, aes(y = Survival_Percentage, x = Time, color = Study)) +

geom_point(aes(x = Time - 0.1 + ifelse(Study == "Registries", -0.05, 0.05), size = Size), alpha = 0.5, position = position_dodge(width = 0.2)) +

geom_segment(aes(x = Time - 0.201 + ifelse(Study == "Registries", -0.05, 0.05), xend = Time + 0.001 + ifelse(Study == "Registries", -0.05, 0.05), y = Survival_Percentage, yend = Survival_Percentage), size = 0.5, position = position_dodge(width = 0.2)) +

geom_segment(aes(x = Time - 0.1 + ifelse(Study == "Registries", -0.05, 0.05), xend = Time - 0.1 + ifelse(Study == "Registries", -0.05, 0.05), y = CI_Lower, yend = CI_Upper), size = 0.5, position = position_dodge(width = 0.2)) +

geom_segment(aes(x = Time - 0.25 + ifelse(Study == "Registries", -0.05, 0.05), xend = Time + 0.05 + ifelse(Study == "Registries", -0.05, 0.05), y = CI_Lower, yend = CI_Lower), size = 0.5, position = position_dodge(width = 0.2)) +

geom_segment(aes(x = Time - 0.25 + ifelse(Study == "Registries", -0.05, 0.05), xend = Time + 0.05 + ifelse(Study == "Registries", -0.05, 0.05), y = CI_Upper, yend = CI_Upper), size = 0.5, position = position_dodge(width = 0.2)) +

scale_size_continuous(range = c(3, 15), breaks = NULL) + # Remove size legend

theme_minimal() +

labs(

y = "Survival Percentage",

x = "Time (years)",

title = "Survival Percentage of Patellofemoral Replacement Over Time",

color = "Study"

) +

theme(panel.grid.major = element_blank(), panel.grid.minor = element_blank()) +

theme(axis.line = element_line(color = "black", linewidth = 0.3))

# Supplementary table 1: outlining the search strategy

| Set | Search Statement |
| --- | --- |
| 1. | (patellofemoral adj2 replace*).mp. [mp=title, abstract, heading word, drug trade name, original title, device manufacturer, drug manufacturer, device trade name, keyword heading word, floating subheading word, candidate term word] |
| 2. | (patellofemoral adj2 arthroplast*).mp. [mp=title, abstract, heading word, drug trade name, original title, device manufacturer, drug manufacturer, device trade name, keyword heading word, floating subheading word, candidate term word] |
| 3. | *patella prosthesis/ |
| 4. | (patello-femoral adj2 replace*).mp. [mp=title, abstract, heading word, drug trade name, original title, device manufacturer, drug manufacturer, device trade name, keyword heading word, floating subheading word, candidate term word] |
| 5. | (patello-femoral adj2 arthroplast*).mp. [mp=title, abstract, heading word, drug trade name, original title, device manufacturer, drug manufacturer, device trade name, keyword heading word, floating subheading word, candidate term word] |
| 6. | 1 or 2 or 3 or 4 or 5 |
| 7. | patello-femoral osteoarthritis.mp. |
| 8. | patellofemoral osteoarthritis.mp. |
| 9. | patello-femoral arthritis.mp. |
| 10. | patellofemoral arthritis.mp. |
| 11. | 7 or 8 or 9 or 10 |
| 12. | (replace* or arthroplast*).mp. [mp=title, abstract, heading word, drug trade name, original title, device manufacturer, drug manufacturer, device trade name, keyword heading word, floating subheading word, candidate term word] |
| 13. | 11 and 12 |
| 14. | 6 or 13 |
